# Activation of TRPV1 Prevents OxLDL-Induced Lipid Accumulation and TNF-****α****-Induced Inflammation in Macrophages: Role of Liver X Receptor ****α****

**DOI:** 10.1155/2013/925171

**Published:** 2013-06-26

**Authors:** Jin-Feng Zhao, Li-Chieh Ching, Yu Ru Kou, Shing-Jong Lin, Jeng Wei, Song-Kun Shyue, Tzong-Shyuan Lee

**Affiliations:** ^1^Department of Physiology, School of Medicine, National Yang-Ming University, Taipei 11221, Taiwan; ^2^Division of Cardiology, Department of Internal Medicine, Taipei Veterans General Hospital, Taipei 11221, Taiwan; ^3^Heart Center, Cheng-Hsin General Hospital, Taipei 11221, Taiwan; ^4^Cardiovascular Division, Institute of Biomedical Sciences, Academia Sinica, Taipei 11529, Taiwan

## Abstract

The transient receptor potential vanilloid type 1 (TRPV1) is crucial in the pathogenesis of atherosclerosis; yet its role and underlying mechanism in the formation of macrophage foam cells remain unclear. Here, we show increased TRPV1 expression in the area of foamy macrophages in atherosclerotic aortas of apolipoprotein E-deficient mice. Exposure of mouse bone-marrow-derived macrophages to oxidized low-density lipoprotein (oxLDL) upregulated the expression of TRPV1. In addition, oxLDL activated TRPV1 and elicited calcium (Ca^2+^) influx, which were abrogated by the pharmacological TRPV1 antagonist capsazepine. Furthermore, oxLDL-induced lipid accumulation in macrophages was ameliorated by TRPV1 agonists but exacerbated by TRPV1 antagonist. Treatment with TRPV1 agonists did not affect the internalization of oxLDL but promoted cholesterol efflux by upregulating the efflux ATP-binding cassette (ABC) transporters ABCA1 and ABCG1. Moreover, the upregulation of ABC transporters was mainly through liver X receptor **α**- (LXR**α**-) dependent regulation of transcription. Moreover, the TNF-**α**-induced inflammatory response was alleviated by TRPV1 agonists but aggravated by the TRPV1 antagonist and LXR**α** siRNA in macrophages. Our data suggest that LXR**α** plays a pivotal role in TRPV1-activation-conferred protection against oxLDL-induced lipid accumulation and TNF-**α**-induced inflammation in macrophages.

## 1. Introduction

Complications of atherosclerosis are the leading cause of death in Western society. Atherosclerosis starts with increased circulating cholesterol levels and involves several events leading to chronic vascular inflammation [1, 2]. The initiation and progression of atherosclerosis largely depend on the function of macrophage foam cells, which focally accumulate within the artery wall and release various cytokines and chemokines to induce inflammation [2–4]. The formation of foam cells primarily results from uncontrolled uptake of modified low-density lipoprotein (LDL) by macrophages, which leads to excess lipoprotein-derived lipid accumulation inside cells and induction of proinflammatory mediators [3, 4]. Cellular lipid levels of foam cells are dynamically regulated by macrophage scavenger receptors (SRs) and cholesterol efflux transporters. The internalization of oxidized LDL (oxLDL) by macrophages is controlled by several types of SRs, including SR-A and CD36. Conversely, cholesterol efflux is mediated by SR-BI and ATP-binding cassette (ABC) transporters such as ABCA1 and ABCG1 [5–10]. Growing evidence indicates that preventing the formation of foam cells can impede the progression of atherosclerosis [11–13], but much remains to be explored.

The transient receptor potential (TRP) vanilloid type 1 (TRPV1), a ligand-gated nonselective cationic channel, is mainly expressed in primary nociceptive sensory neurons [14, 15]. Neuronal TRPV1 is activated by heat, protons, endogenous lipid molecules and oxidative stimuli [14–19] and exogenous agonists such as evodiamine (an active ingredient of the evodia fruit) and capsaicin (an active ingredient of hot pepper) [16, 20]. Activation of TRPV1 permits calcium (Ca^2+^) entry, which results in an elevated level of intracellular Ca^2+^ and serves as a Ca^2+^ signal to elicit responses in neurons [14, 15].

TRPV1 expresses in certain types of nonneuronal cells such as epithelial and endothelial cells and regulates several of their pathophysiological functions [21–24]. Emerging evidence suggests that TRPV1 may be an important sensor and regulator of cardiovascular homeostasis and a protector against certain cardiovascular diseases such as hypertension, endothelial dysfunction, and stroke [25, 26]. Recently, we reported that deletion of TRPV1 worsened atherosclerotic lesions in apolipoprotein E-deficient mice (ApoE^−/−^, a model of atherosclerosis-prone mice), so TRPV1 may have a role in atherogenesis [27]. However, the expression of TRPV1 in macrophages is still unclear and its functional significance in macrophage foam cells remains largely unknown.

In this study, we aimed to address the role of TRPV1 in the pathophysiological functions of macrophage foam cells. We first examined TRPV1 expression in macrophages and its distribution in mouse atherosclerotic lesions, then, investigated the therapeutic effects of 2 TRPV1 agonists, evodiamine, and capsaicin, on the deregulation of lipid metabolism and inflammation in macrophages. Finally, we explored the molecular mechanisms underlying the protection conferred by TRPV1 agonists in macrophages.

## 2. Materials and Methods

### 2.1. Reagents

Evodiamine, human LDL, capsaicin, capsazepine, apolipoprotein AI (apoAI), high-density lipoprotein (HDL), EGTA, Oil-red O, and mouse antibody for *α*-tubulin were from Sigma-Aldrich (St. Louis, MO, USA). Mouse antibody for TRPV1 was from Abnova (Taoyuan, Taiwan). Rabbit antibodies for ABCG1, CD36, histone H1, goat antibody for SR-A, control small interfering RNA (siRNA), and LXR*α* siRNA were from Santa Cruz Biotechnology (Santa Cruz, CA, USA). Mouse antibody for ABCA1, *α*-actin, and rabbit antibodies for SR-BI, LXR*α*, and F4/80 were from Abcam (Cambridge, MA, USA). Macrophage colony-stimulating factor (MCSF), tumor necrosis factor *α* (TNF*α*), and ELISA kits were from R&D systems (Minneapolis, MN, USA). Dil-labeled oxLDL was from Biomedical Technologies (Stoughton, MA, USA). NBD-cholesterol and T0901317 were from Cayman Chemical (Ann Arbor, MI, USA). The Fluo-8 Ca^2+^ assay kit was from ABD BioQuest (Sunnyvale, CA, USA). Cholesterol and triglyceride assay kits were from Randox (Crumlin, Co. Antrim, UK).

### 2.2. Mice

The investigation conformed to the Guide for the Care and Use of Laboratory Animals by the US National Institutes of Health (NIH Publication No. 85-23, revised 1996), and all animal experiments were approved by the Animal Care and Utilization Committee of National Yang-Ming University. Wild-type (WT) C57BL/6 mice were purchased from the National Laboratory Animal Center, National Science Council (Taipei)

### 2.3. Cell Culture

Bone-marrow-derived macrophages (BMDMs) were prepared as described [28]. Briefly, WT mice were killed by CO_2_ exposure, and mononuclear cells from femurs were harvested by Percoll (1.073 g/cm^3^) density gradient centrifugation. The cells were seeded in minimum essential medium *α* (MEM*α*) supplemented with 50 ng/mL MCSF, 10% fetal bovine serum (FBS), and penicillin (100 U/mL)/streptomycin (100 g/mL) at 37°C for 5 d. Mouse macrophage-like J774.A1 cells (Bioresource Collection and Research Center; Hsinchu, Taiwan) were cultured in RPMI 1640 medium supplemented with 10% FBS, penicillin (100 U/mL), and streptomycin (100 *μ*g/mL).

### 2.4. LDL Modification

LDL was exposed to CuSO_4_ (5 *μ*mol/L) for 24 h at 37°C and Cu^2+^, then, removed by extensive dialysis. The extent of modification was determined by measuring thiobarbituric acid-reactive substances (TBARs). OxLDL containing approximately 30–60 nmol of TBARs defined as malondialdehyde equivalents per milligram LDL protein was used for experiments.

### 2.5. Western Blot Analysis

BMDMs were rinsed with PBS, then, lysed in immunoprecipitation lysis buffer (50 mmol/L Tris pH 7.5, 5 mmol/L EDTA, 300 mmol/L NaCl, 1% Triton X-100, 1 mmol/L phenylmethylsulfonyl fluoride, 10 *μ*g/mL leupeptin, and 10 *μ*g/mL aprotinin). Aliquots (50 *μ*g) of cell lysates were separated on 8% SDS-PAGE. After transfer to membranes, samples were immunoblotted with primary antibodies, then, horseradish peroxidase-conjugated secondary antibodies. Bands were revealed by use of an enzyme-linked chemiluminescence detection kit (Perkin Elmer, Waltham, MA, USA), and density was quantified by use of ImageQuant 5.2 (Healthcare Bio-Sciences, Philadelphia, PA, USA).

### 2.6. Measurement of [Ca^2+^]_*i*_ Level

Ca^2+^ assay was performed according to the manufacturer's protocol (ABD BioQuest, Sunnyvale, CA, USA). Briefly, BMDMs were seeded in 24-well plates and grown for 24 h. Cells were then washed and Fluo-8 NE dye-loading solution was added for 1 hr at room temperature. Medium was then replaced with fresh medium containing test compounds. Fluorescence was measured by fluorometry (Molecular Devices, Sunnyvale, CA, USA) with 490 nm excitation and 525 nm emission.

### 2.7. Oil-Red O Staining

Cells were fixed with 4% paraformaldehyde and then stained with 0.5% Oil-red O. Hematoxylin was used for counterstaining.

### 2.8. Dil-OxLDL Binding Assay

Dil-oxLDL, labeled with green fluorescence, has been used to measure oxLDL binding to SRs of macrophages [29]. Briefly, BMDMs were treated with concentrations of evodiamine or capsaicin for 24 h, then, incubated with Dil-labeled oxLDL (10 *μ*g/mL) for an additional 4 h at 4°C. After a washing with phosphate-buffered saline (PBS), cell lysates were analyzed by fluorometry (Molecular Devices, Sunnyvale, CA, USA) at 540 nm excitation and 590 nm emission.

### 2.9. Cholesterol Efflux Assay

BMDMs were treated with concentrations of evodiamine or capsaicin for 12 h, then, underwent equilibration with NBD-cholesterol (1 *μ*g/mL) for an additional 6 h. NBD-cholesterol-labeled cells were washed with PBS and incubated in MEM*α* for 6 h with apoAI (10 *μ*g/mL) or HDL (50 *μ*g/mL). Fluorescence-labeled cholesterol released from cells into the medium was measured by use of a multilabel counter (PerkinElmer, Waltham, MA, USA) at 485 nm excitation and 535 nm emission. Cholesterol efflux was expressed as percentage fluorescence in the medium relative to total fluorescence (cells and medium).

### 2.10. Preparation of Nuclear Extracts

Nuclear extracts were prepared as described [30]. BMDMs were lysed in 10 mM Hepes pH 7.9, 10 mM KCl, 1.5 mM MgCl_2_, 0.5% Nonidet P-40, 1 *μ*g/mL leupeptin, 10 *μ*g/mL aprotinin, and 1 mM phenylmethylsulfonyl fluoride. Nuclei were pelleted at 5000 g  for 5 min at 4°C, and the resulting supernatant was used as the cytosolic fraction. Nuclei were resuspended in lysis buffer, sheared for 15 sec by microprobe sonication, and incubated on ice for 5 min. After centrifugation at 12000 g  for 5 min at 4°C, supernatant was collected as the nuclear extract.

### 2.11. Chromatin Immunoprecipitation (ChIP)

ChIP assays were performed as described [12]. BMDMs were cultured in MEM*α* with or without pretreatment with evodiamine (0.5 *μ*M) or capsaicin (10 *μ*M) for 6 h and fixed by formaldehyde for 15 min at room temperature. After cells were lysed and sonicated, chromatin solution was diluted and cells were incubated overnight with rabbit anti-LXR*α* Ab or rabbit IgG at 4°C. Immunocomplexes were precipitated with salmon sperm DNA/protein A agarose and collected by centrifugation. After cells were washed, chromatin DNA was eluted, purified, and subjected to PCR analysis. An amount of 1% chromatin solution was used as an input control. The mouse *ABCA1 *gene promoter containing LXR binding element was amplified by PCR with the following primer sequences: 5′-CCA CGT GCT TTC TGC TGA GT-3′ and 5′-TGC CGC GAC TAG TTC CTT TT-3′. PCR products were resolved on a 2% agarose gel and visualized by ethidium bromide staining.

### 2.12. Transient Transfection and Luciferase Reporter Assay

Cells were transfected with the plasmids phABCA1 (-928)-Luc, a reporter plasmid for the human ABCA1 promoter with LXR*α* responsive element (LXRE, 3′-AAACTGGC TATCATTGGA GACGCG-5′) or phABCA1-DR4 m-Luc, a reporter plasmid with a mutation in the LXRE (3′-AAACACAC TATCATTGAT GACGCG-5′), by use of TurboFect. The pGL3-renilla plasmid was cotransfected as a transfection control. After transfection for 24 h, cells were treated with evodiamine (500 nM), capsaicin (10 *μ*M), or T0901317 (10 *μ*M), an LXR agonist, for another 24 h. Cells were then lysed for Luc and renilla activity assays. 

### 2.13. Small Interfering RNA Transfection

Macrophages were transfected with control or LXR*α* siRNA with use of TurboFect for 24 h and then treated with evodiamine or capsaicin for another 24 h before further experiments. 

### 2.14. Measurement of Inflammatory Cytokines

The levels of proinflammatory cytokines, including monocyte chemoattractant protein-1 (MCP-1), interleukin-6 (IL-6), and macrophage inflammatory protein-2 (MIP-2), in culture medium were measured by use of ELISA kits.

### 2.15. Statistical Analysis

Results are presented as mean ± SD from 5 independent experiments. Mann-Whitney test was used to compare 2 independent groups. The Kruskal-Wallis test followed by Bonferroni post-hoc analysis was used to account for multiple testing. SPSS v20.0 (SPSS Inc., Chicago, IL) was used for analysis. Differences were considered statistically significant at *P* < 0.05.

## 3. Results

### 3.1. Expression of TRPV1 in Macrophages and Atherosclerotic Lesions

**Figure 1:**
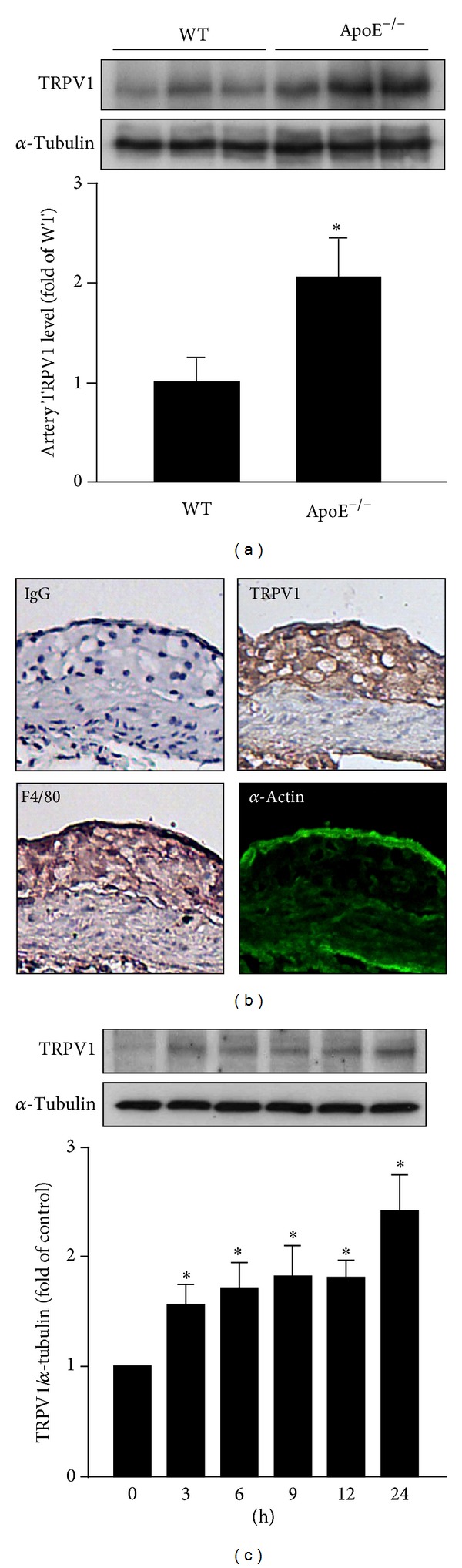
Expression of TRPV1 is increased in atherosclerotic lesions of ApoE^−/−^ mice. (a) Western blot analysis of protein expression of TRPV1 in aortas from 5-month-old ApoE^−/−^ and wild-type (WT) mice. *α*-Tubulin was a normalization control. Data are mean ± SD from 6 animals. *P < 0.05 versus WT mice. (b) Immunohistochemical staining for TRPV1, F4/80 (macrophage marker), and *α*-actin (smooth-muscle-cell marker) in atherosclerotic lesions of aortas from 5-month-old ApoE^−/−^ mice. Specificity of immunostaining was confirmed with an IgG-negative control. Hematoxylin was used as counterstaining. Magnification = 100 x. (c) Western blot analysis of protein expression of TRPV1 induced by oxLDL (50 *μ*g/mL) relative to that induced by vehicle (PBS) for 0–24 h. Data are mean ± SD from 5 independent experiments. *P < 0.05 versus vehicle-treated group. *α*-Tubulin was a normalization control.

To study the possible role of TRPV1 in atherogenesis, we first investigated the expression of TRPV1 in atherosclerotic lesions. The protein level of TRPV1 was markedly higher in ApoE^−/−^ than wild-type mouse aortas (Figure 1(a)). In addition to the expression of TRPV1 in aortic ECs, immunohistochemical staining for TRPV1 demonstrated positive signals confined mainly to areas of macrophages in atherosclerotic lesions of ApoE^−/−^ mice (Figure 1(b)). Because neuronal TRPV1 can be activated by several oxidative stimuli and lipids [14, 18, 19, 24], we next examined the effect of oxLDL on the expression of TRPV1 in macrophages. Treating BMDMs with 50 *μ*g/mL oxLDL for up to 24 h time-dependently increased the expression of TRPV1 (Figure 1(c))   as early as 3 h after treatment or up to 24 h. Thus, TRPV1 may play an important role in the development of atherosclerosis.

### 3.2. OxLDL Upregulates and Activates TRPV1 in BMDMs

**Figure 2:**
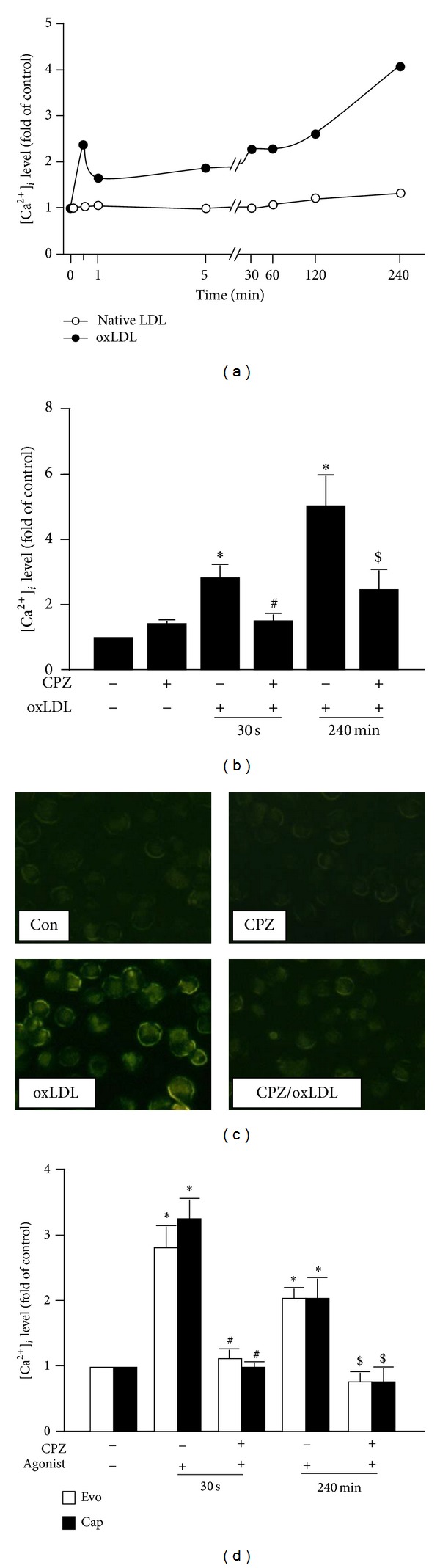
Treatment with oxLDL upregulates and activates TRPV1 in macrophages. (a) Intracellular levels of Ca^2+^ ([Ca^2+^]_*i*_) in response to incubation with oxLDL (50 *μ*g/mL) or native LDL (50 *μ*g/mL). [Ca^2+^]_*i*_ was quantified by measuring the intensity of Ca^2+^-sensitive Fluo-8 fluorescence. (b) [Ca^2+^]_*i*_ level at 30 sec and 240 min after incubation with oxLDL in BMDMs pretreated or not with capsazepine (CPZ; 10 *μ*M). Data are mean ± SD from 5 independent experiments. *P < 0.05 versus vehicle, ^#^ *P* < 0.05 versus 30 sec/oxLDL, and ^$^ *P* < 0.05 versus 240 min/oxLDL. (c) Representative microscopy images of Ca^2+^-binding Fluo-8 fluorescence at 240 min after incubation with or without oxLDL in BMDMs pretreated or not with capsazepine. (d) [Ca^2+^]_*i*_ level at 30 sec and 240 min after incubation with evodiamine (Evo; 0.5 *μ*M) or capsaicin (Cap; 10 *μ*M) in BMDMs pretreated or not with capsazepine. Data are mean ± SD from 5 independent experiments. *P < 0.05 versus LDL-treated group or vehicle, ^#^ *P* < 0.05 versus 30 sec/Evo- or Cap-treated group, and ^$^ *P* < 0.05 versus 240 min/Evo- or Cap-treated group.

We next investigate the stimulatory effect of oxLDL on the channel activity of TRPV1 in BMDMs. In response to oxLDL, the intracellular level of Ca^2+^ ([Ca^2+^]_*i*_) in BMDMs, as reflected by intensity of Ca^2+^-sensitive Fluo-8 fluorescence, rapidly peaked at 30 sec, slightly decreased at 1 min, and gradually increased to peak again at 4 h (Figure 2(a)). Importantly, the oxLDL-induced increase in [Ca^2+^]_*i*_ level at 30 sec and 4 h poststimulation were prevented by pretreatment with capsazepine (a TRPV1 antagonist) (Figures 2(b) and 2(c)). We then checked the specificity of capsazepine and found that exposure of BMDMs to evodiamine or capsaicin (TRPV1 agonists) also increased [Ca^2+^]_*i*_ level at 30 sec, which was abolished by capsazepine pretreatment (Figure 2(d)).

### 3.3. Activation of TRPV1 by Agonists Suppresses Lipid Accumulation in Macrophage Foam Cells

**Figure 3:**
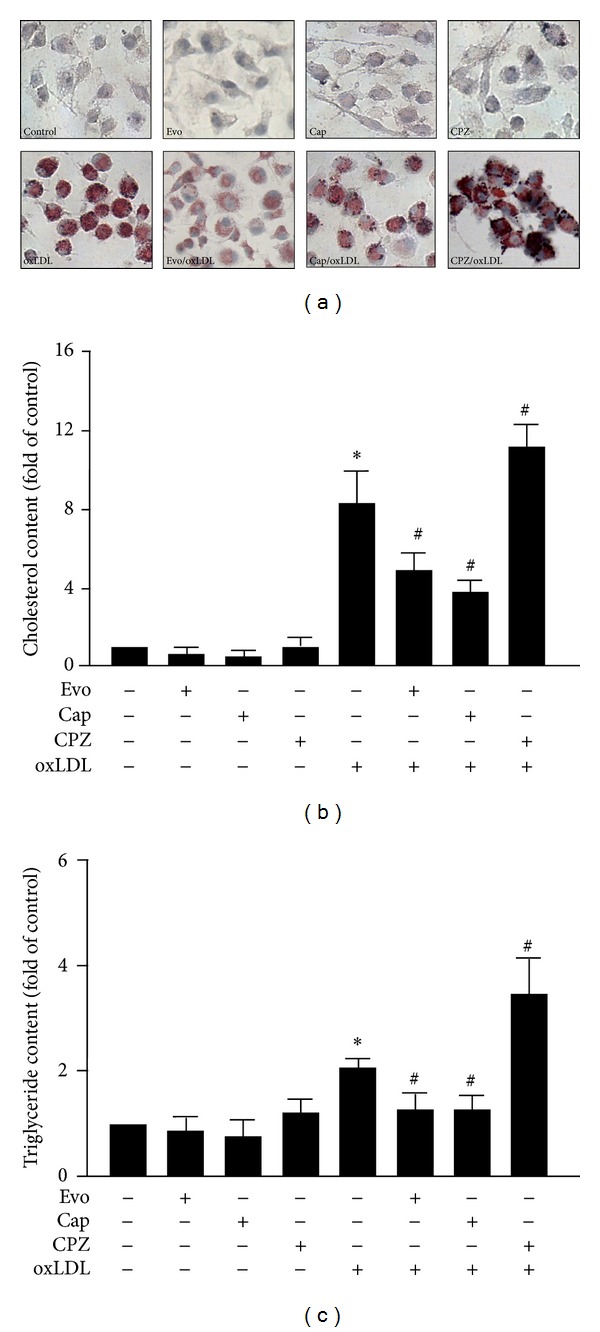
Activation of TRPV1 by agonists alleviates oxLDL-induced foam-cell formation.Cells were incubated with vehicle (DMSO), evodiamine (0.5 *μ*M), capsaicin (10 *μ*M), or capsazepine (10 *μ*M) with or without oxLDL (50 *μ*g/mL). (a) Representative microscopy images of cells with intracellular lipids stained by Oil-red O. Hematoxylin was used as counterstaining. Magnification  =  400 x. ((b) and (c)) Intracellular levels of cholesterol (b) and triglycerides (c) were extracted by use of hexane/isopropanol (3/2, v/v) and analyzed by colorimetric assay kits. Data are mean ± SD from 5 independent experiments. *P < 0.05 versus vehicle-treated cells, ^#^ *P* < 0.05 versus oxLDL-treated cells.

We then determined the functional significance of TRPV1 in foam-cell formation in BMDMs. Pretreatment with evodiamine or capsaicin decreased oxLDL-induced lipid accumulation, as revealed by Oil-red O staining (Figure 3(a)) and cellular levels of cholesterol and triglycerides (Figures 3(b) and 3(c)). In contrast, capsazepine treatment augmented oxLDL-induced lipid accumulation (Figures 3(a)–3(c)).Thus, activation of TRPV1 by agonists may protect against foam-cell formation.

### 3.4. Activation of TRPV1 by Agonists Enhances Cholesterol Efflux without Altering OxLDL Internalization

**Figure 4:**
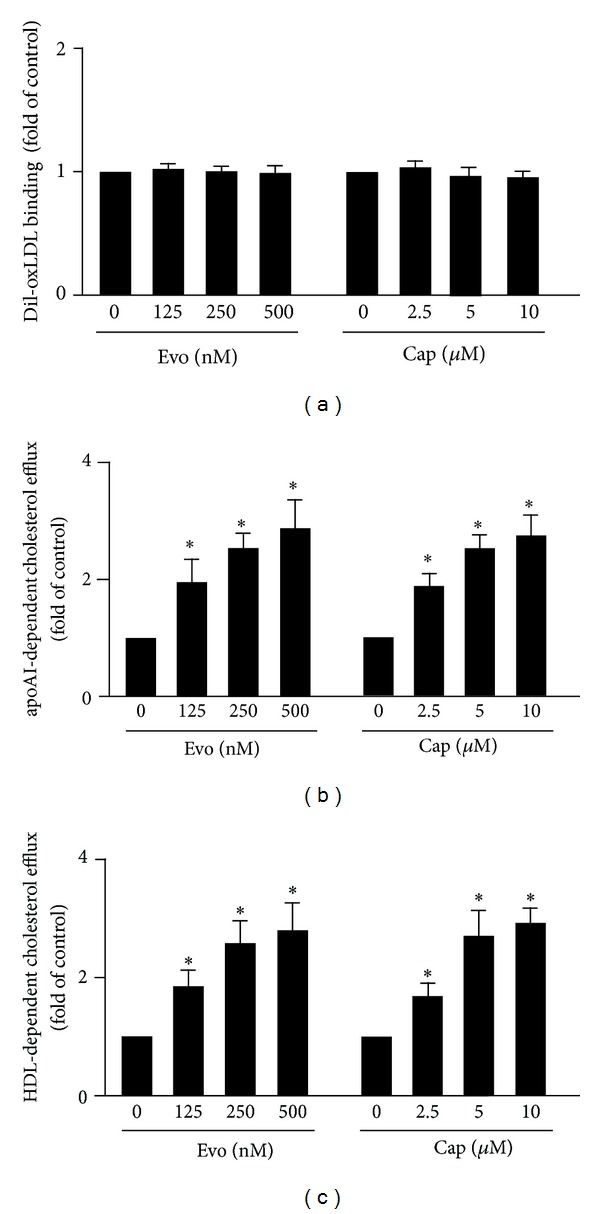
TRPV1 activation by agonists promotes apoAI- and HDL-dependent cholesterol efflux in macrophages. (a) For Dil-oxLDL binding assay, BMDMs were treated with vehicle, evodiamine (125, 250, 500, 500 nM), or capsaicin (2.5, 5, 10 *μ*M) for 12 h and then incubated with 10 *μ*g/mL Dil-oxLDL at 4°C for 4 h. Cellular lysates were analyzed by fluorimetry. ((b) and (c)) BMDMs were treated with indicated concentrations of evodiamine (125, 250, 500, 500 nM) or capsaicin (2.5, 5, 10 *μ*M) for 12 h, followed by NBD-cholesterol (1 *μ*g/mL) for another 6 h in the presence of (b) apoAI (10 *μ*g/mL) or (c) HDL (50 *μ*g/mL). The medium and cell lysates were collected for the measurement of fluorescence. Cholesterol efflux was defined as fluorescence in the medium relative to total amount of fluorescence. Data are mean ± SEM from 5 independent experiments. *P < 0.05 versus vehicle treatment.

**Figure 5:**
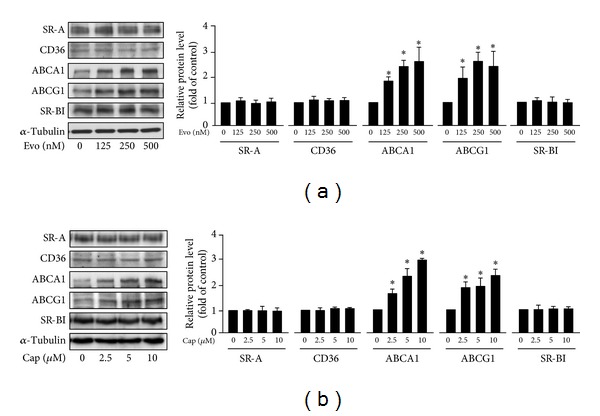
Effect of TRPV1 activation on expression of SR-A, CD36, SR-BI, ABCA1, and ABCG1 in macrophages. BMDMs were incubated with vehicle, (a) evodiamine (125, 250, 500 nM), or (b) capsaicin (2.5, 5, 10 *μ*M) for 24 h. Western blot analysis of protein levels of SR-A, CD36, ABCA1, ABCG1, SR-BI, and *α*-tubulin. Data are mean ± SD from 5 independent experiments. *P < 0.05 versus vehicle-treated cells.

We then elucidated the effect of TRPV1 agonists on oxLDL internalization and cholesterol efflux. Pretreating BMDMs with the TRPV1 agonists evodiamine (0.5 *μ*M) or capsaicin (10 *μ*M) did not alter the cholesterol binding but dose-dependently increased the apoAI- or HDL-dependent cholesterol efflux (Figures 4(a)–4(c)). SR-A, CD36, SR-BI, ABCA1, and ABCG1 have crucial roles in cholesterol homeostasis during foam-cell formation [5–10]. We therefore delineated the mechanisms of TRPV1 agonists in attenuating lipid accumulation by examining the alteration in expression of these receptors and transporters. Macrophages treated with evodiamine or capsaicin showed increased protein levels of ABCA1 and ABCG1, with no change in protein levels of SR-A, CD36, and SR-BI (Figures 5(a) and 5(b)). Therefore, TRPV1 activation suppressing intracellular lipid accumulation is likely due to an increase in reverse- cholesterol- transporter- (RCT-) dependent cholesterol efflux but not inhibition of SR-mediated oxLDL uptake in macrophages.

### 3.5. LXR*α* Mediates the Suppressive Effect of TRPV1 Agonists on Foam-Cell Formation

**Figure 6:**
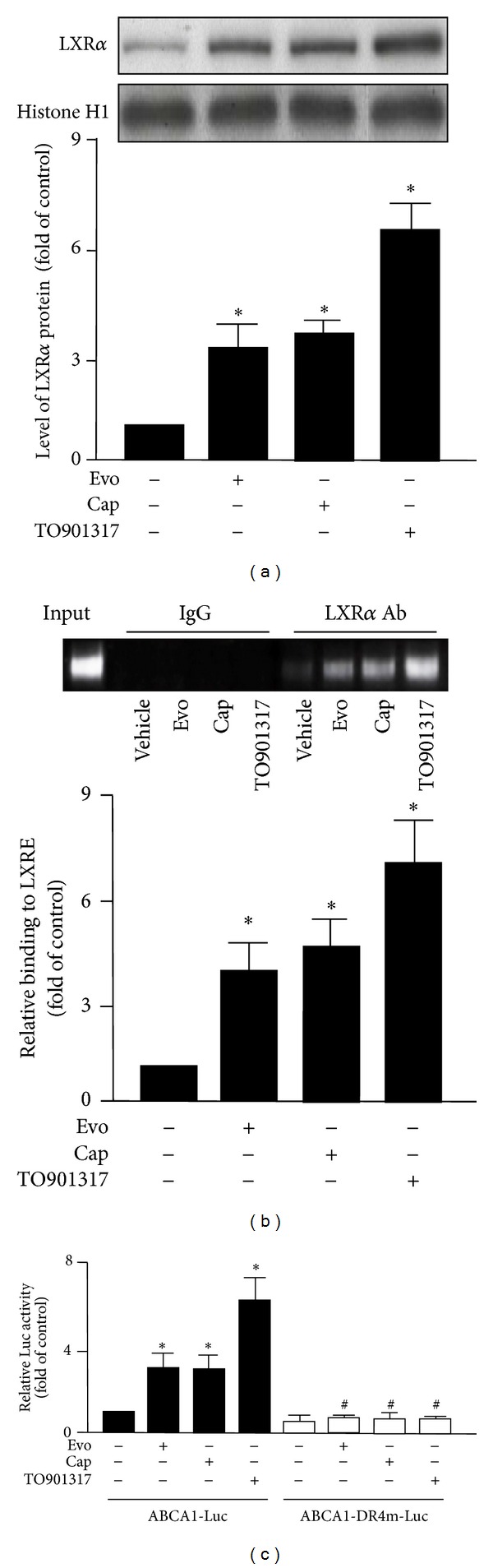
Treatment with TRPV1 agonists increases the activation of LXR*α* in macrophages. (a) BMDMs were pretreated with capsazepine (10 *μ*M) for 1 h then incubated with evodiamine (500 nM), capsaicin (10 *μ*M), or T0901317 (10 *μ*M) for 6 h. Western blot analysis of nuclear protein level of LXR*α* and Histone H1 as a normalization control. (b) Macrophages were treated with vehicle, evodiamine (500 nM), capsaicin (10 *μ*M), or T0901317 for 6 h, then immunoprecipitated with anti-LXR*α* or rabbit IgG. PCR amplification involved specific primers for the ABCA1 gene promoter. The amplified DNA products were separated by electrophoresis with 2% agarose gel. (c) Macrophages were transfected with plasmid phABCA1-Luc or phABCA1-DR4 m-Luc for 24 h, treated with vehicle, evodiamine (500 nM), capsaicin (10 *μ*M), or TO901317 (10 *μ*M) for 24 h, then lysed for Luc activity assays with renilla activity as an internal control. Data are mean ± SD from 5 independent experiments. *P < 0.05 versus vehicle-treated cells, ^#^ *P* < 0.05 versus phABCA1-Luc-transfected cells with evodiamine or capsaicin treatment.

**Figure 7:**
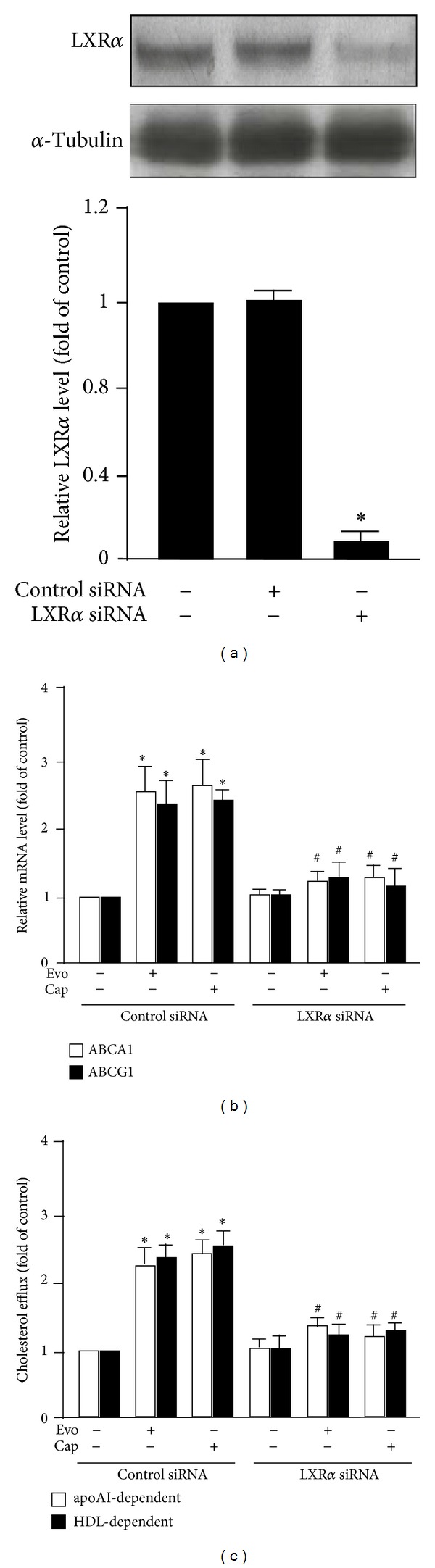
Knockdown of LXR*α* abolishes the protein expression of ABCA1 and ABCG1 and attenuates lipid accumulation by TRPV1 agonists. BMDMs were preincubated with control siRNA (50 nmol/L) or LXR*α* siRNA (50 nmol/L) for 24 h, followed by evodiamine or capsaicin treatment for additional 24 h. (a) Western blot analysis of protein expression of LXR*α*. (b) RT-PCR analysis of mRNA expression of ABCA1 and ABCG1. (c) ApoAI- and HDL-dependent cholesterol efflux was evaluated by use of NBD-cholesterol. Data are mean ± SD from 5 independent experiments. *P < 0.05 versus control siRNA-treated cells, ^#^ *P* < 0.05 versus control siRNA-treated cells with evodiamine or capsaicin treatment.

To address whether the LXR*α* is involved in TRPV1-agonist-induced expression of ABCA1 and ABCG1, we examined the nuclear protein level of LXR*α* in evodiamine- or capsaicin-treated macrophages. Evodiamine, capsaicin, or T0901317 (LXR*α* agonist) treatment increased the nuclear level of LXR*α* (Figure 6(a)) and enhanced binding of LXR*α* to LXRE in the ABCA1 promoter (Figure 6(b)). Furthermore, to explore the transcriptional regulation of LXR*α* in evodiamine-treated macrophages, we performed LXR activation assays by transfecting with phABCA1 (-928)-Luc or phABCA1-DR4 m-Luc (reporter plasmid with a mutation in LXRE), followed by evodiamine or capsaicin treatment. As compared with the control group, treatment with evodiamine, capsaicin, or T0901317 as a positive control markedly increased the promoter activity of phABCA1 (-928)-Luc (Figure 6(c)). In contrast to phABCA1-Luc, phABCA1-DR4 m-Luc showed blunted induction with evodiamine, capsaicin, or T0901317 treatment (Figure 6(c)). In addition, transfection with LXR*α* siRNA decreased the protein expression of LXR*α* and abolished the mRNA expression of ABCA1 and ABCG1 induced by evodiamine or capsaicin (Figures 7(a) and 7(b)). Moreover, siRNA inhibition of LXR activation abrogated the beneficial effect of evodiamine or capsaicin on apoAI- or HDL-dependent cholesterol efflux (Figure 7(c)). These results indicate the essential role of LXR*α* activation in evodiamine- or capsaicin-regulated gene expression of ABCA1 and ABCG1, which may contribute to the suppressive effect of the agonists in the transformation of macrophage foam cells.

### 3.6. Knockdown of LXR*α* Abolishes TRPV1 Activation-Conferred Protection in the TNF-*α*-Induced Inflammatory Response

**Figure 8:**
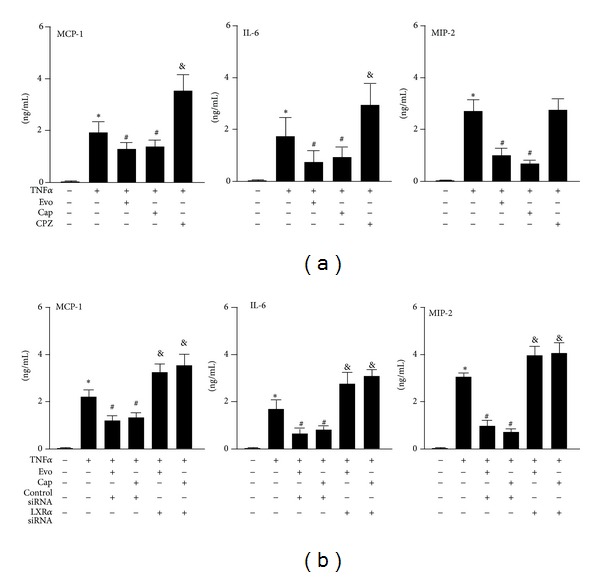
Knockdown of LXR*α* diminishes the protective effect of TRPV1 agonists against TNF-*α*-induced inflammation in macrophages. (a) BMDMs were pretreated with capsazepine (10 *μ*M) for 1 h or (b) transfected with control siRNA (50 nmol/L) or LXR*α* siRNA (50 nmol/L) for 24 h, then incubated with vehicle (DMSO), evodiamine (500 nM), and capsaicin (10 *μ*M) with or without TNF-*α* (10 ng/mL) for an additional 18 h. ELISA of levels of MCP-1, IL-6, and MIP-2 in the culture medium. Data are mean ± SD from 5 independent experiments. *P < 0.05 versus vehicle-treated cells, and ^#^ *P* < 0.05 versus TNF-*α*-treated cells, and ^&^ *P* < 0.05 versus TNF-*α*-treated group with evodiamine or capsaicin treatment.

TNF-*α* is a key proatherogenic mediator for the progression of atherosclerotic lesions [31]. We next examined the effect of TRPV1 agonists on the TNF-*α*-induced inflammatory response in macrophages. TNF-*α*-increased production of MCP-1, IL-6, and MIP-2 in BMDMs was significantly attenuated by pretreatment with the 2 TRPV1 agonists; moreover, pretreatment with capsazepine exacerbated the TNF-*α*-induced production of MCP-1 and IL-6 but not MIP-2 (Figure 8(a)). Additionally, evodiamine or capsaicin suppressing the TNF-*α*-induced increase in MCP-1, IL-6, and MIP-2 production was reversed by siRNA inhibition of LXR*α* activation (Figure 8(b)). These results suggest that LXR*α* activation is required for the anti-inflammatory action of TRPV1 agonists in macrophages.

## 4. Discussion

Here we characterized a new effect of TRPV1 activation and its underlying molecular mechanism in suppressing oxLDL- or TNF-*α*-induced deregulation of lipid metabolism and inflammation in macrophages. We first validated TRPV1 expression in atherosclerotic aortas and in particular regions of macrophage-foam cells. The accumulation of macrophage-derived foam cells in the intima and subsequent release of inflammatory cytokines from these cells are 2 critical steps in the initiation and progression of atherosclerosis [1–4]. This cellular localization implies the possible role of TRPV1 in regulating the pathophysiological functions of such cells. We thus used an *in vitro* model to study the role of TRPV1 in macrophage-foam cells. Incubation with evodiamine or capsaicin, TRPV1 agonists, alleviated the oxLDL-induced lipid accumulation and TNF-*α*-induced inflammation in BMDMs, so the function of TRPV1 is linked to the lipid metabolism and inflammatory response of macrophage-foam cells. Interestingly, the protective effects of TRPV1 agonists may be due to the activation of LXR*α*. Our *in vitro* data suggest that TRPV1 has a novel effect in maintaining lipid homeostasis and the inflammatory response in macrophages. 

We then investigated the molecular mechanisms underlying the beneficial function of TRPV1 activation in macrophages by use of this experimental cell culture model. Treatment with oxLDL, the most important modulator in the development of atherosclerosis, increased TRPV1 channel activity in BMDMs, as evidenced by a TRPV1-mediated increase in [Ca^2+^]_*i*_ level to a profile similar to that evoked by TRPV1 agonists. In addition, oxLDL-induced foam-cell formation, as evidenced by increased cellular levels of cholesterol and triglycerides, was suppressed by TRPV1 agonists but exacerbated by a TRPV1 antagonist. Removal of extracellular Ca^2+^ by EGTA aggravated the oxLDL-induced lipid accumulation and abrogated the protective effect of TRPV1 agonist in BMDMs (data not shown), which agrees with previous studies that the increase in [Ca^2+^]_*i*_ level induced by oxLDL may play a key role in the formation of macrophage-foam cells [32]. Therefore, activation of TRPV1/Ca^2+^ signaling may inhibit the formation of foam cells* in vitro*.

SR-dependent oxLDL uptake and RCT-mediated cholesterol efflux are 2 key regulatory mechanisms in the intracellular lipid homeostasis of macrophage-foam cells [5–10]. Several lines of evidence indicate that reduced expression of SRs or elevated function of RCTs in macrophages leads to reduced deposition of cholesterol in macrophages [12, 30, 33]. Interestingly, TRPV1 agonist treatment did not alter the binding of Dil-oxLDL to SRs or the protein expression of SR-A, CD36, and SR-BI in BMDMs but promoted cholesterol efflux. Moreover, TRPV1 agonist treatment upregulated both ABCA1 and ABCG1, 2 major types of ABC transporters responsible for cholesterol efflux from macrophage-foam cells to apoAI and HDL, respectively. The critical role of ABCA1 and ABCG1 in maintaining cholesterol homeostasis in macrophages has been well defined [34, 35]. Loss or impaired function of ABCA1 or ABCG1 in human or experimental rodents leads to hyperlipidemia, excessive cholesterol accumulation in peripheral tissues, and an overwhelming inflammatory response [34, 36]. Thus, our *in vitro* results strongly support that the TRPV1-mediated suppression of foam-cell formation was solely due to an increase in RCT-dependent cholesterol efflux, which is consistent with the previous studies that cytokine- or flavonoid-induced upregulation of ABCA1 or ABCG1 contributes to alleviated lipid accumulation in foam cells [11–13]. The detailed mechanism by which activation of TRPV1 leads to upregulation of ABC transporters is not clear. However, an increase in [Ca^2+^]_*i*_ level evoked by other interventions may regulate the expression of ABC transporters in macrophages [37]. 

Additionally, we showed that the TRPV1 agonist-induced upregulation of ABCA1 and ABCG1 was accompanied by an increase in nuclear levels of LXR*α* and its DNA binding ability. This notion is further supported by findings that TRPV1-agonist-induced increase in promoter activity was abrogated by transfection with the LXRE mutant (phABCA1-DR4 m-Luc). Inhibition of LXR*α* activation by siRNA diminished the TRPV1-agonist-mediated upregulation of ABCA1 and ABCG1. Thus, LXR*α*-mediated transcriptional regulation may be required for induction of ABCA1 and ABCG1 expression by TRPV1 agonists. Although we found a unique pathway for TRPV1 activity, the detailed molecular mechanisms of TRPV1 agonists affecting cholesterol efflux merit further investigation. In functional analysis to inhibit LXR*α* activation, the suppressive effect of TRPV1 agonists on intracellular lipid accumulation was abolished. LXR*α* may be required for the TRPV1 activation-induced gene expression of ABCA1 and ABCG1, which may contribute to suppressing the transformation of macrophage foam cells *in vitro*. In addition to the LXR*α*-mediated transcriptional regulation, increasing evidence suggests that ABCA1 is also regulated by the posttranscriptional modification [38, 39]. For example, calmodulin is known to prevent protein degradation of ABCA1 by interacting with calmodulin-binding motif (1244 to 1257 amino acids within ABCA1 protein), which is located near the PEST sequence (1283 to 1306 amino acids) and thus prevents the binding of calpain to ABCA1, leading to the inhibition of ABCA1 degradation [38]. However, whether the posttranscriptional regulation is engaged in the TRPV1 agonist-mediated upregulation of ABCA1 remain the further investigations.

Emerging evidence suggests that in addition to its action on cholesterol metabolism, ABCA1 also functions as a critical modulator in regulating the inflammatory response [39, 40]. Patients with Tangier disease and ABCA1 mutation or mice with functional ablation of ABCA1 show an irregular inflammatory response [41–43]. Moreover, growing evidence demonstrates that the expression and activity of ABCA1 is impaired during inflammation *in vivo* [44, 45]. Treatment with pro-inflammatory cytokines or lipopolysaccharide (LPS) decreases the expression of ABCA1 and its related function in various types of cells [44, 45]. More importantly, viral or bacterial infection decreases the expression of LXR*α* and that of its target genes, including ABCA1 [46]. In addition, activation of LXR*α* by its ligands inhibits the LPS-induced production of pro-inflammatory mediators including TNF-*α*, MCP-1, IL-6, and MIP-2 in macrophages [46–48]. We found that TNF-*α*-induced production of MCP-1 and IL-6 was inhibited by treatment with TRPV1 agonists but augmented by the TRPV1 antagonist. siRNA knockdown of LXR*α* expression abolished the anti-inflammatory effect of TRPV1 agonists on TNF-*α*-treated BMDMs. Activation of TRPV1 by its agonists also suppressed the inflammatory response of macrophages *in vitro*, which both may explain the protective role of TRPV1 in reducing atherosclerotic lesions *in vivo *[49–51]. Conceivably, the anti-inflammatory property of LXR*α* may play a pivotal role in the crosstalk between reverse cholesterol transport and immunity. However, the detailed molecular mechanism underlying this interaction needs further investigation.

TRPV1 is originally found expressed in primary nociceptive sensory neurons and plays an important role in detecting irritative, inflammatory, and oxidative substances by somatic and visceral afferents [14, 15]. However, increasing evidence suggests that TRPV1 is expressed in several types of non-neuronal cells, including macrophages [52], endothelial cells (ECs) [27], and preadipocytes [53, 54] and vitally regulates their functions. Recently, convergent sets of evidence support a physiological role for TRPV1 as a crucial integrator in the functions of the cardiovascular system and in cardiovascular diseases [25, 27, 55]. For example, TRPV1 activation by capsaicin increased the activity of endothelial NO synthase (eNOS), promoted vasorelaxation, and thereby reduced blood pressure in experimental hypertensive rats [25]. Additionally, we previously reported that TRPV1 activation by evodiamine or capsaicin triggered Ca^2+^-dependent PI3 K/Akt/CaMKII/AMPK signaling, thus leading to eNOS activation in ECs [27, 56]. As well, inactivation of TRPV1 accelerates the development of metabolic disorders such as hypertension, atherosclerosis, obesity, and fatty liver, whereas TRPV1 agonist activation protects against these metabolic diseases [25, 55, 57, 58]. Our previous study demonstrated that chronic treatment of ApoE^−/−^ mice with the TRPV1 agonist evodiamine alleviated hyperlipidemia, systemic inflammation, hepatic macrovesicular steatosis, and atherosclerosis [59]. Moreover, our recent data demonstrated that chronic treatment with evodiamine upregulated the hepatic levels of LDLR, ABCG5, ABCG8, and CYP7A1 in ApoE^−/−^ mice, which may promote bile acid synthesis and fecal excretion. Interestingly, activation of LXR*α* potentiated cholesterol excretion by increasing the transcriptional regulation of LDLR, ABCG5, ABCG8, and CYP7A1 in liver [60]. These lines of evidence indicate the potential role of TRPV1 in lipid metabolism. This notion is substantiated by our findings that activation of TRPV1 by evodiamine increased [Ca^2+^]_*i*_ level, which activated the LXR*α* signaling pathway and led to the upregulation of ABCA1 and ABCG1, ultimately reducing the lipid accumulation of macrophage-foam cells. Altogether, although the target cells for evodiamine have not yet been identified, our studies strongly suggest that collaboration of multiple physiological pathways in different types of cells may be required for the beneficial effects of TRPV1 agonists in treating metabolic disorders.

## 5. Conclusions

In conclusion, we demonstrate a novel protective function of TRPV1 in macrophages, whereby activation by TRPV1 agonists could suppress the oxLDL-induced deregulation of lipid metabolism and inflammation *in vitro* by an increase in LXR*α* activation, thus leading to the promotion of cholesterol efflux and attenuation of the inflammatory response. Our findings may help in developing novel pharmacological targets for treating atherosclerosis-related cardiovascular diseases.
